# Generative models of morphogenesis in developmental biology

**DOI:** 10.1016/j.semcdb.2023.02.001

**Published:** 2023-09-30

**Authors:** Namid R. Stillman, Roberto Mayor

**Affiliations:** a Department of Cell and Developmental Biology, University College London, Gower Street, London WC1E 6BT, UK; b Center for Integrative Biology, Faculty of Sciences, Universidad Mayor; Santiago, Chile Santiago, Chile.

**Keywords:** Developmental biology, Cell migration, Computational biology, Generative models, Simulation-based inference

## Abstract

Understanding the mechanism by which cells coordinate their differentiation and migration is critical to our understanding of many fundamental processes such as wound healing, disease progression, and developmental biology. Mathematical models have been an essential tool for testing and developing our understanding, such as models of cells as soft spherical particles, reaction-diffusion systems that couple cell movement to environmental factors, and multi-scale multi-physics simulations that combine bottom-up rule-based models with continuum laws. However, mathematical models can often be loosely related to data or have so many parameters that model behaviour is weakly constrained. Recent methods in machine learning introduce new means by which models can be derived and deployed. In this review, we discuss examples of mathematical models of aspects of developmental biology, such as cell migration, and how these models can be combined with these recent machine learning methods.

## 1 Introduction

Mathematical models have been a central tool in the search for laws underpinning biological organisms, at least as far back as Galileo, and possibly as far back as Aristotle [1]. These models have been used to test hypotheses, plan experiments, and conceptualise life at the smallest length scales. However, as the rate of data generation has increased, due to increased compute power and advanced microscopy techniques, the role of mathematical models has changed. Traditional biophysical models such as those developed using equations and assumptions of Newtonian mechanics, statistical physics, or hydrodynamics simplify biological objects into minimal systems described by parameters which relate to biologically meaningful properties including the stiffness of a cell or the cortical tension of its membrane [2], [3], [4]. These models are developed to be sparse, such that they contain a small number of parameters, typically fewer than ten and almost always fewer than hundreds. These models are then verified by comparing model output, such as the speed and/or persistence time of a single cell or cellular cluster, with experiment observations. These simplified models of biology are not expected to describe all the complexities of the phenomenon of interest, but only those aspects which are thought to be fundamental to the underlying causal mechanism giving rise to the phenomenon. As such, they are well suited as a conceptual tool for describing mechanism and planning down-stream experiments.

Simultaneously, the era of big data and compute has introduced a new family of mathematical models which are extremely well suited to high dimensional and multi-modal data but significantly less interpretable. Deep learning models, which make use of artificial neural networks (NNs), have been shown to be adept at many tasks including image classification, translation, grid-based game playing, and text-to-image generation. These models have hundreds of parameters at a minimum, but typically hundreds of thousands, if not millions. Furthermore, these variables are not, *prima facie*, related to biological or physical features within the data. While downstream methods can be used to correlate model parameters with observable features, such as cell shape or fluorescence intensity [5], [6], these methods for interpretability are typically only partially sufficient, such that the biological or physical interpretation of most model parameters remain inaccessible. Despite, or perhaps because of, the poor interpretability of these models, they can be extremely powerful at pattern recognition and discovery. For example, NNs are now standardly used for segmentation and tracking of cells in timelapse microscope data [7], [8].

Both deep learning and traditional mathematical models appear to be two distinct tools that serve biological inquiry. On the one hand, biophysical models are highly interpretable, are developed to have as few parameters as possible, and are applied to relatively low dimensional data or, at least, summarising features of high-dimensional data. On the other hand, deep learning models work with high dimensional data and a very large number of parameters. These models achieve high accuracy at many tasks with little to no domain knowledge while sacrificing interpretability for statistical power. However, as we shall describe, both modelling paradigms can be understood under the broader framework of generative modelling. Generative models seek to model the underlying stochastic procedure for generating data, opposed to discriminate models which seek to separate data into sub-classes or categories. When understood as generative models, both approaches share many similarities and can, in principle, be combined. We posit that doing so would allow for interpretable models of high-dimensional data or, alternatively, low-dimensional models that can provide highly accurate predictions of experiment observations.

In this paper, we describe how biophysical models have been used in developmental biology and describe some of their limitations as traditional forms of generative models. We describe biophysical models of three processes fundamental to developmental biology: cell-cell interactions, collective cell migration, and physical morphogenesis. We then describe generative models that use neural network models, so-called deep generative models. We set out the main forms of deep generative models and provide recent examples of their use. As a nascent field, we are limited by the number of applications of deep generative models to developmental biology specifically, and instead describe the application of deep generative models to cell biology more broadly. We additionally set out how both traditional and deep generative methods might be combined as a form of hybrid models that both retain the explainability of biophysics and the flexibility of deep learning models. Finally, we conclude with some future perspectives on the role of generative models in cell biology.

## 2 Biophysics models

**Fig. 1:**
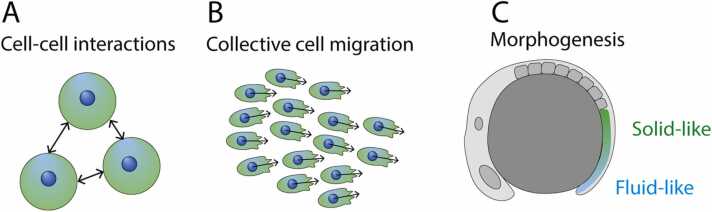
Mathematical models across length scales. We give examples of three phenomena at increasing length-scales where mathematical models are often used including at the scale of (a) single cells, where cell-cell interactions are modelled as repulsive and adhesive forces, (b) cell clusters which migrate with coordinated motion across distances larger than a single cell radius, and (c) morphogenesis which involves the interaction of both chemical and mechanical cues.

The application of physics-based models to biology has provided significant insights into the mechanisms that underpin many biological processes. These biophysical models make explicit assumptions on the balance of forces on cell sheets, single cells, or intracellular dynamics. The models are in constant development, with new models constantly being proposed to account for newly observed phenomenon. As such, a comprehensive review will always be insufficient to fully account for the many active avenues of research. For example, we do not address recent models of bioelectrical signalling, recently described for observations in both morphogenesis and regeneration, as reviewed in [9]. Similarly, we do not fully detail models for gene-regulatory networks (GRN) which have been reviewed in, for example, [10]. Instead, we describe a relatively small subset of mathematical models as related to three fundamental processes of development biology: cell-cell interactions, collective cell migration, and physical models of morphogenesis (Fig. 1). We note that these three processes are not disjoint but rather describe features of an organism’s development from increasingly large length-scales. Hence, each model can rely, in part, on the subsequent model and this reasoning has motivated interest in multi-scale models to more fully account for these coupled coarse-grain levels of enquiry. In this review, we consider each level in isolation and refer the interested reader to the following reviews specific to multi-scale models [11], [12], [13]. Finally, we do not to describe the relative strengths or weaknesses of specific biophysics modelling frameworks, for which much has been written elsewhere, as in [14], [15], and where the success of a model should partially be judged on a case-by-case basis, but instead choose to discuss the broader limitations of traditional biophysics models and neural networks-based models in Section 4.

### 2.1 Models of interactions between cells

Understanding how cells maintain rheological states, such as confluent tissue or co-ordinated fluid migration, involves understanding effects at length-scales both larger and smaller than that of a single cell. For example, the rheology of cellular collectives is known to be influenced both by the number (or packing fraction) of cells within a region as well as the adhesion or stiffness by which a cell pulls or pushes on its nearest neighbour. These forces, exerted on and by cells, are a result of cell-cell and cell-substrate interactions and these, in turn, are due to the complex interplay of different molecules and signalling pathways. Hence, understanding the collective dynamics of cells requires multi-scale models that include a highly simplified model of cellular interactions.

We assume that models of natural phenomena aim to reproduce at least some subset of observable features from a system of interest. For example, a model of cell-cell interactions might seek to, as accurately as possible, describe the impact of cells pushing and pulling on observables such as the position and/or velocity of neighbouring cells [16]. This model is likely to be simplified as the known mechanism by which cells push and pull depends on nanoscale surface components, such as membrane receptors, and on cytoskeletal dynamics at the interface of two or more cells. Yet, the model might also account for known changes in migratory behaviour that results in changes in interactions. The complex dynamic between the cell, its environment, and its internal cytoskeleton results in three broad classes of migratory modes: *amoeboid, epithelial,* and *mesenchymal*. These modes are transient, whereby cells can transition between, for example, epithelial to mesenchymal modes (undergoing the so-called epithelial-mesenchymal transition or EMT) or from mesenchymal to amoeboid (the so-called mesenchymal-amoeboid transition or MAT) [17], [18], [19], [20]. A sheet of epithelial cells is typically characterised by strong and long-lasting tight junctions between cells, such as E-cadherin, whereas mesenchymal clusters have been observed to migrate with weaker bonds between cells, such as N-cadherin. For further information about both modes of migration and the relevant molecular junctions, see for example [21]. More recently, amoeboid cells have also been observed to act as a collective, and whereas the interactions between amoeboid collectives is unclear, it is expected that cell-cell interactions for amoeboid clusters are significantly weaker than even mesenchymal [22]. The observation that even amoeboid cells, which are thought to interact weakly, can exhibit coordinated collective behaviours, demonstrates that cells have a high sensitivity to forces exerted by their neighbours and substrate. Despite the identification of different migratory phenotypes, it remains unclear how these modes arise from changes at the molecular basis of cell-cell interactions, such as in E- and N-cadherin expression, and how this leads to changes in collective states.

Whereas the molecular basis for many cell-cell interactions has been well characterised, an appropriate biophysics model of cell-cell interactions is far from complete. Cell-cell interactions can be modelled as a pair-potential function which describes the energy, and hence forces, experienced by each cell as a function of distance [23], [24]. Repulsive (pushing) forces are applied when cell volumes overlap, for example, whereas adhesive (pulling) forces might be introduced within some pre-defined radius of interaction. Modelling cell-cell interactions as pair potential has led to realistic simulations for many observed phenomena including contact-inhibition of locomotion (CIL), the influence of actin-cables on supracellular systems, cell-cell alignment and flocking, and cell jamming transitions [25], [26], [27], [28]. These models assume that all cells interact as soft or stiff spheres, borrowing potential functions from those in colloidal sciences [29], and that the potentials are constant and uniform across the cellular system. Hence, only one or two parameters, such as the strength of attractive and repulsive forces, are needed to describe all interactions between cells.

There is clear evidence that mechanical forces between cells are known to be critical for both tissue homeostasis and in initiating collective behaviours such as migration in scratch assays [30], [31]. Furthermore, interactions between cells are known to be highly heterogenous both across different migratory models (epithelial/mesenchymal), within migratory modes (epithelial), and across time (initiation of leader cells during wound healing). Hence, it is often remarkable that many mathematical models can recapitulate observations using a simple, uniform, and constant description of cell-cell interactions. A more complete understanding of interactions might extend one of the mathematical potentials used to model cellular forces to account for cellular heterogeneity. Interactions would then be described by a pair-potential that varies across a population and, possible, in time. Identifying the parameters for this heterogenous and dynamic potential would require careful coupling of models to cell assays and may benefit from hybrid approaches. In Section 4, we set out an illustrative example of how this might be achieved.

### 2.2 Models of collective cell migration

Cell-cell interactions can only be understood in the context of multi-cellular systems. While single cells can change their migratory behaviour based on environmental factors, such as the local chemical, material, or geometric properties, cell collectives additionally alter their migratory behaviour based on interactions with their nearest neighbours. The cellular collective can then be biased towards certain directional cues and this collective migration can be more persistent than the directionality of the single cell. For example, bacterial populations that migrate as a collective can move more persistently due to a ‘collective smoothing’ of their perturbations at the scale of single cells [32]. Additionally, cells have been observed to migrate across a gradient of surface tension within the collective, taking advantage of surface phenomena such as the Marangoni effect [33].

Models of cell collectives incorporate both cell-cell interactions with models of self-propulsion and other cellular forces. For example, a model of cells as soft spheres, as described above, will typically include an active force and some stochasticity when modelling cell collectives, so-called active Brownian particle models [34], [35]. Alternatively, the model might start with a description of the membrane of cells as a confluent sheet and resolve the elasticity and tension in the cell membrane, assuming a preferred area or volume for each cell, leading to so-called active vertex models [36], [37]. Choices of which modelling framework to use, as well as which forces, and the dimensionality of the model will be driven largely by the mechanism under investigation as well as the available experimental data.

Models of collective cell migration have been part motivated by the observations that cellular sheets share many of the micro-scale properties of materials and that rheological models can be used to describe systems of cells [29], [38], [39], [40]. For example, cellular systems have been well described as a viscoelastic material and this viscoelasticity is related to cellular rearrangements, division rates, and rigidity clusters [41], [42], [43]. These models involve both a description of the interaction between cells, modelled as springs or as soft spheres, as well as the active propulsion force that differentiates the trajectories of living cells from random Brownian motion. Under this view, cellular collectives become a form of *active matter* where the material properties do not reach steady state due to the self-propulsion (active) force of single cells but where the material has a determinable rheology. Identifying the parameters that describe cellular active matter, as relevant to experimental observations, is often calculated from point estimates of summary statistics, such as the slope of the mean square displacement or mean and variance of the distribution of velocities of cells, which are then compared to simulation output. In Section 3, we outline other recent methods for parameter inference of these systems.

As cell density increases, cells can become jammed or packed such that the structural properties resulting from the collective dominate over effects from single cell dynamics. This transition has been referred to as the jamming or glassy transition and simulated using both active Brownian and vertex models [44], [45], [46], [47]. Furthermore, these models have provided predictions for when this transition occurs, for example as a function of the structural order or packing fraction of the system, which have been verified in certain systems [48], [49], [50]. These phase transitions in cellular active matter, such as the jamming transition, highlight how rheological changes can arise from coordinated collective dynamics. However, how a transition induces a specific rheology remains an open question, even for inactive systems such as soft microgel particles [51].

The ability to predict the conditions for material phase transitions is important for understanding conditions that lead to the proper functioning of biological tissue. For example, identifying the conditions by which cells become more fluid or solid was used to explain the antero-posterior axis elongation in zebrafish [52]. For a review on the role of phase transitions in physical tissue, see, for example [53]. However, understanding cells as undergoing a glassy transition, for example, is in part hindered by the absence of a complete theory of glassy matter and non-equilibrium matter. Hence, models of cells as active matter typically use point estimates of summary statistics such as the exponent of the mean square displacement or average velocity to match simulations to observations and look for hallmarks of glassy behaviour such as cells becoming crowded, changes to the mean square displacement, and heterogeneity in their motion [50], [53], [54]. Chief among the challenge of understanding cells as active matter is the similar challenge in glassy physics, namely how we relate changes in the structural order of a system to the collective dynamics [55], [56], [57]. Recent research on understanding glass transitions *in silico* has highlighted the strength of recent advances in machine learning tools, such as in [58], and we later make the argument that these tools are fitting for quantifying living matter too. The combination of statistical physics with machine learning methods might allow for detailed rheological models coupled with chemical processes of the cell. These multi-physics models are already being deployed, for example in understanding morphogenesis.

### 2.3 Models of morphogenesis

At larger length-scales, models of morphogenesis seek to reproduce the form and structure of a specific stage of embryo development [59]. These models combine models of collective cell migration and cell-cell interactions with chemical cues that spread over the cellular system by coupling discrete models of cell motion with, for example, reaction-diffusion or hydrodynamic equations. Hence, morphogenesis models are typically multi-physics, contain more parameters and can be computationally expensive to simulate. Furthermore, these models also depend implicitly on complex gene-regulatory networks (GRN) which require their own specific modelling approaches, as in, for example. [60], [61].

Models of morphogenesis can combine multiple complementary models of developmental biology. For example, the combination of growing tissue with signalling pathways has been shown to reproduce certain patterning on, for example, catfish shark [62]. Indeed, many morphogenesis models use models of pattern formation such as reaction-diffusion equations that lead to Turing patterns. These models have been used to describe features ranging from striped patterning in *Drosophila melongaster* embryos, somite formation and vertebrate limb bud development [63], [64], [65], [66], [67]. At sufficient scale, these models can average across discrete representations of cells to describe instead the developing tissue as a continuum with relevant constituent equations taken from continuum mechanics [68], [69], [70]. These coarse-grain description of cell migration highlights the power of rheological descriptions of cells at multiple length scales.

Furthermore, these models allow for the inclusion of several complementary cell types within a system. For example, embryonic development involves the cells which vary not just in spatial and mechanical composition but also in biological function such as tissue cells and immune cells [71]. Accounting for heterogenous cellular populations requires distinct modelling frameworks, in part due to their distinct migratory modes. For example, tissue cells are typically more epithelial/mesenchymal and immune cells are more amoeboid. Hence, models of morphogenesis are sometimes required to combine several models at smaller length scales [72], [73]. This again highlights the interconnectedness and multi-scale requirement for comprehensive models for developmental biology, where models across length-scale should be consistent.

Whereas the requirement for consistency across length scales can be a challenge, especially for models that aim to combine multiple observations of the behaviour of single cells into models of several hundreds of thousands, this can also provide an opportunity for the role of mathematical models [74]. Recent advances in synthetic biology and organoid technologies allows for high-precision control over the design of biological environments and cell culture [75], [76], [77], [78]. For example, cells cultured in three dimensional aggregates and with physiologically realistic extracellular matrix can be combined with a vasculature that introduces hydrodynamic effects in a semi-controllable fashion [79], [80]. These *in vitro* models provide large data output for fitting mathematical models which seek to explain the compounding effects of multiple bio-physical phenomena [81], [82]. The challenge in the future is expected to be in fitting these models whereby the number of parameters and observations can be considerably large.

## 3 Deep generative models

In the preceding sections, we have described examples where biophysics has been used to model an underlying process as accurately as possible. Typically, model accuracy is evaluated by comparing model output to some specific observables or summary statistics. In this sense, biophysical models can be understood as describing a generative process, namely a process which leads to the generation of a measurable quantity, where we measure the system and/or computational output using summary statistics [83], [84]. Generative models are distinct from discriminative models which separate data into subsets, for example through a classification or clustering algorithm. Instead, generative models aim to describe the entire dataspace and the underlying data generating process [85].

**Fig. 2:**
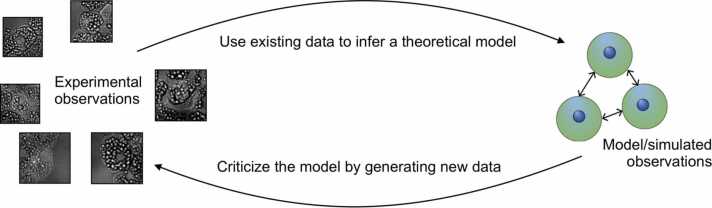
Lifecycle of a model. We provide a schematic of Box’s loop, in which models are generated by inferring from data and then criticised by generating new data to test model hypotheses.

By describing biophysical models as generative models, we gain insight into the main process by which a model might be developed. First, we observe some specific behaviour or phenotype of interest. We then attempt to develop a model that can reproduce this behaviour in as simple a system as possible, such that the model can reproduce only observed behaviours rather than observed and opposing. We might seek models with as few unfixed parameters as is possible to construct. With both model and experiment observations, we would then fit the remaining unfixed parameters using our experiment observations. Assuming that we have been able to estimate the required parameters and our model can reproduce observed behaviours, we can use our model to predict new behaviours of the system and suggest new experiments. This lifecycle of a model is well described by [86] and set out schematically in (Fig. 2) for the case of cell biology.

**Fig. 3:**
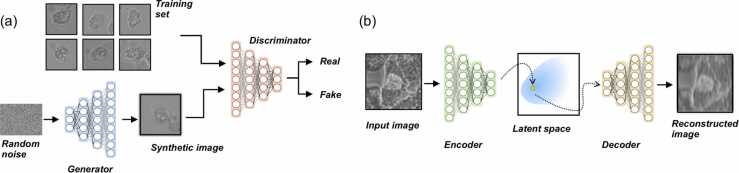
Examples of deep generative models. In (a) we give the schematic for the generative adversarial network (GAN) which is a type of implicit model, whereas in (b) we show an autoencoder (AE) architecture which is explicit, as the latent space can be directly sampled from. When the latent space is composed of probability densities, this architecture is known as variational autoencoder (VAE).

As described, biophysics models are generative, in that they seek to describe the underlying generative process of the phenomenon and require observations and parameters to assess the robustness and validity of the model. These models share similarities with deep generative models, an active sub-class of machine learning models, which also seek to describe the data generative process but using artificial neural networks. These models typically have many thousands or hundreds of thousands of parameters and do not rely on the same biological and physics-based intuition as biophysics models. These deep generative models are also more expressive, such that they can reproduce more complex phenomenon than traditional generative models, are much better at processing high dimensional datasets, and are typically much less interpretable. Yet, they can also be combined with biophysics models to estimate parameters, model error, or unknown model components. In what follows, we give an overview of some recent deep generative models that have been applied to cell biology. Examples of both implicit and explicit model architectures is shown in (Fig. 3).

### 3.1 Implicit generative models

**Fig. 4:**
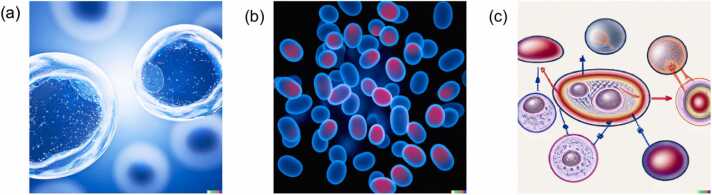
Generating images from text prompts. We demonstrate three example images generated using the DALL-E2 model from OpenAI, using the prompts (a) “cell-cell interactions”, (b) “collective cell migration”, and (c) “cell and developmental biology”.

Deep generative models use artificial neural networks to approximate the data generating process. These models can then be used to generate new synthetic data which is similar to the data used for training the artificial neural networks. Models that learn only the stochastic procedure used for generating data are known as implicit models and include generative adversarial networks (GANs) as well as the more recent denoising diffusion models which have shown extremely strong performance at generating, for example, text-to-image models that can generate highly expressive images from text prompts [87], [88], [89]. See (Fig. 4) for example images generated from text prompts.

Implicit models such as GANs have been used extensively in cell biology in recent years to aid computer vision tasks such as segmenting and tracking cells [90], [91]. Furthermore, given the success of recent diffusion models, we expect that there will be a similar increase in diffusion-based models being used to aid image analysis such as to predict 3D cell geometries from 2D images [92]. While this will almost certainly allow for new and exciting avenues of research, implicit models are particularly prone to limitations of interpretability as the learnt generative process is typically not straightforwardly accessible [93], [94].

### 3.2 Explicit generative models

Rather than learning only the stochastic procedure, explicit generative models learn to reproduce the underlying data distribution as accurately as possible and this learnt representation, often referred to as the latent space, is sampled from to be able to generate new synthetic examples. As a result, the learned distribution can be probed to identify key features that represent changes in data or combined with other methods to, for example, estimate parameters [95].

One of the most popular explicit models is the autoencoder (AE) and the probabilistic extension, the variational autoencoder (VAE). Both AEs and VAEs follow a bottleneck-like approach to approximating the dataspace, whereby artificial neural networks learn to encode the input data into a low dimensional space, the latent space, and a decoder learns to take samples from the latent space and turn them back into the input data as closely as possible. This is done by minimising the difference between input data (that is passed to the encoder) and the output data (which decodes samples from the latent space) and, for VAEs, ensuring that the latent space is represented by an appropriate probability density which can be sampled from. Recent work has demonstrated that VAEs can be used to not only generate new synthetic data in cell biology but, crucially, that the latent space also corresponds to biophysical properties relating to, for example, cell cycle or metastatic properties of carcinoma cells [5], [96].

Explicit models often seek to model the entire data space in terms of probability densities. These probabilistic models include both AEs, and VAEs, as well as normalising flows (NFs) which learn to generate samples by performing a series of nonlinear transformations on the distribution of data [97], [98]. By learning probability densities, explicit models can also be used for tasks other than data generation. One of the recent applications of explicit models is in model inference, where simulations are combined with deep generative models to better estimate parameter uncertainty or model misspecification.

Known more broadly as either simulation-based inference or likelihood-free inference, these methods use artificial neural networks, such as NFs, to approximate the probability density of model parameters, conditioned on experiment observations, to give information on which parameter sets are most likely, given some observations [95], [99], [100]. SBI is similar to previous parameter estimation techniques such as approximate Bayesian computation. However, the benefit of SBI methods is the application of neural networks which improves the efficiency and accuracy of the inference, as well as increasing both the dimensionality of the summary statistics used to estimate parameters, as well as the number of parameters. This is seen in, for example, [101], which included a 37-parameter system of neural dynamics. SBI and explicit models more broadly have yet to be used extensively in cell biology.

## 4 Hybrid generative models

The above description of deep generative models demonstrates their applicability for computer vision tasks, such as segmentation, and some initial research on applying explicit generative models to inferring biologically relevant data. Furthermore, SBI methods provide a means for better coupling simulation data with experiments and provides a new framework by which we can better estimate whether parameters and models accurately reproduce experiment observations. However, generative models might be used to infer biophysics models directly from data and, under certain conditions, biophysics can be combined with artificial neural networks so that model components are directly learned from data rather than through assumptions by the model developer.

To develop models directly from data, explicit generative models can be combined with methods for inferring symbolic equations. For example, symbolic regression has been applied to an explicit generative model (a VAE) of a simulation of interacting particles and the system was able to correctly identify the equations for the interaction between particles, such as being connected by springs or by gravitational forces and gave a proposed equation for astronomical bodies [102]. While this approach has yet to be applied to interacting biological objects, such as cells, doing so could provide new insights. Alternatively, artificial neural networks have been used to augment model equations where only partial dynamics are known but full model output is available, as in [103] which demonstrated that such a system can predict missing forces in, for example, a reaction-diffusion system, even when only partial model equations are provided. Again, this work has yet to be applied to problems in biology but could have impact on systems where multiple physical processes are known to take place, such as during morphogenesis, to approximate unknown force terms. Finally, recent work has shown that neural networks can learn an entire cell simulator, and this learnt simulator is able to reproduce simulation output several orders of magnitude faster than the original simulator [104]. These hybrid and generative approaches are expected to increase in the future and point towards a new era in AI-assisted biophysics research.

To better understand how these hybrid and generative models might be used in practice, it is useful to consider an illustrative example. Suppose several timelapse videos of cell migration are obtained with the aim of identifying the effect of a molecular knock-out experiment on cell behaviours. The first aim is to reproduce, as faithfully as possible, salient features of the control group. This requires a biophysics model which might be, for example, constructed using a cellular automata or particle-based framework. To couple model to data, simulation-based inference can then be used, where parameters such as the active force, persistence timescale, or interaction strength are inferred from measures such as the average speed of the cluster or their mean square displacement. We might then suppose that the inferred posterior, describing the certainty of parameters given the experiment observations, shows a high uncertainty on the parameters describing the cell-cell interactions.

To improve upon the model and address this uncertainty, the interaction potential in the model might be replaced with a neural network which learns to predict forces from both experiment and simulation data. This neural network is passed the metric distance between cells as input and returns the physical forces as output. The simulation model is now a hybrid model that contains both biophysics (encoded in modelling framework) and a ‘black box’ (represented by the neural network). If simulation-based inference is applied again, it might then find that the active force and persistence timescale has much higher certainty and the model output better matches observations. Finally, we might want to understand how the interactions are affected by the knock-out experiment. To do so, we could apply symbolic regression to the neural network to retrieve a closed-form symbolic equation with few parameters (opposed to the high-parameter NN). These derived symbolic equations can be interrogated alongside experiments to link changes in collective dynamics to molecular dynamics or returned to simulation-based inference to further identify uncertainty within the hybrid model.

The above example makes explicit how hybrid models can address the tension between models that rely on ‘black-box’ (NN) and ‘white-box’ (biophysics) components. A NN-based model can identify system features, including cell phenotypes such as their metastatic potential, from over-parameterised models and large amounts of e.g., image data. However, unlike biophysics models, they are blind to what aspects of cell biology that are used to build this inference. For example, a VAE trained on image data will learn associations not just based on cell-geometries but also image quality, lighting, and experimental noise. Hybrid models overcome this by replacing singular aspects of a biophysics model (such as cell-cell interactions) with a NN-approximator, restricting the aspects that the black-box components are required to learn. Given this restricted domain, we can then combine NNs with symbolic regression to untangle the inferred dependences into something that can be interpreted such as an equation for distance-based cellular forces. Hence, hybrid learning resolves the tension between modelling paradigms by restricting the application of NNs to a subset of the broader biology under investigation.

## 5 Future perspectives

We present the above discussion as a high-level overview on recent advances in machine learning and their relevance to models of cell biology more broadly and developmental biology specifically. Mathematical models have been a crucial part in building and advancing our understanding of biological processes, through the development of simplified models which can be used to test predictions and promote new hypothesis. However, the ubiquity of cheap computing means that it is now easier than before to develop complex simulations that reproduce, in part, observations of the relevant biological process. This is advantageous in that we can produce models faster but comes at the cost of increasing model complexity without corresponding certainty in results. Models designed for specific observed behaviours can have multiple parameters with high uncertainty that reproduce a multitude of conflicting behaviours. Alternatively, researchers might purposefully restrict the number of parameters within a model to avoid ‘overfitting’ but use broad system measures, such as the speed of a cellular collective, which weakly constrains the fitting procedure. Finally, these models can, at times be used to support experimental observations as evidence, rather than to drive new hypotheses or as tools for conceptual understanding. This is especially problematic where models are developed *post hoc* to reproduce experiment observations and become purpose built for a specific set of experiments, making them narrowly defined and weakly generalizable.

The advent of machine learning methods does not, *prima facie*, present a means for resolving the tension of how models are used or abused in biology. Indeed, many machine learning methods have been used to infer models of GRN directly from data, for example in [105], [106]. However, in this paper we are specifically interested in the role of machine intelligence in identifying mechanistic or causal models in developmental biology. Deep generative models, especially those that allow for explicit formulation of the learned representation, provides a way to better couple models to data in a way that makes it much more difficult to use models as evidence and identify low-level mechanisms. This might be achieved by using SBI methods to estimate parameter uncertainty more robustly and to make use of all the data when calibrating models, as we set out in Section 3. Moreover, hybrid models combine NNs with biophysics models to learn individual model components directly from data. In this context, NNs learn specific features for the biophysics models which has the effect of both restricting the domain for black-box inference and increasing their explainability. Additionally, this allows biophysics models to incorporate, for example, experiment noise or cellular heterogeneity through the NN components without having to explicitly state these effects. Furthermore, by identifying which (physical) model components or parameters are observed to be similar across different model systems and multiple independent observations, we can improve our understanding of whether such components accurately represent biophysical properties and are not artefacts of the modelling framework or a specific experimental set-up. For example, a collective effort to identify cell-cell interaction forces, as relied upon in many biophysics model frameworks, could be performed across multiple different research groups and cellular systems to isolate common features that would underpin future modelling approaches.

We believe that many future findings in developmental biology will likely come from increased collaboration between biologists, physicists, and experts in machine intelligence. As our theoretical understanding increases and we identify more causal mechanisms, recent advances in synthetic biology and cell engineering means that we can better build and break these mechanisms through precise experiment design [107]. In this work, we focus predominately on the future of model building in developmental biology but refer the reader to recent advances in synthetic engineering, such as morphogenesis, for further details [108], [109], [110].

The final balance between machine learning and biophysics will likely take time to resolve but we overview both fields to foster collaboration and highlight future opportunities. Given that machine learning, and especially deep learning, methods are typically viewed as ‘black box’ approaches that are not straightforwardly amenable to identifying causal mechanisms but able to perform well on large data and given that biophysics models are typically well-coupled to physical processes but applied to low dimensional data such as point estimates of a system (the average speed of a cell cluster), then the possible benefits of combining both methods seem clear. At the same time, we note that the integration of distinct frameworks can be a considerable challenge, both culturally and technically. To do so requires a shared theoretical language and awareness of current and outstanding theoretical questions. Developing both will also take time. However, we believe that cell and developmental biology is well placed to foster this collaborative spirit, given the interdisciplinary exchange between biologists and biophysicists to date. Hence, we look forward to shared future research that uncovers more of how we understand life at the smallest length scales and how we might integrate advances in machine intelligence to do so.

## Conflict of interest

No conflict of interest.
